# Taking lead from the community: What do young people living with HIV want us to research?

**DOI:** 10.1371/journal.pgph.0002605

**Published:** 2023-12-11

**Authors:** Arish Mudra Rakshasa-Loots, Kaylee S. van Wyhe, Shalena Naidoo, Ntuthu Daizana, Barbara Laughton, Tembela Boyana

**Affiliations:** 1 Family Centre for Research with Ubuntu (FAMCRU), Department of Paediatrics and Child Health, Stellenbosch University, Cape Town, South Africa; 2 Edinburgh Neuroscience, School of Biomedical Sciences, The University of Edinburgh, Edinburgh, United Kingdom; 3 ACSENT Lab, Department of Psychology, University of Cape Town, Cape Town, South Africa; The University of Texas Health Science Center at Houston School of Public Health - San Antonio Campus, UNITED STATES

## Abstract

Priority setting workshops enable researchers to take the lead from people with relevant lived experience, and design research which authentically responds to community needs. Large-scale global priority setting exercises have previously identified key research questions related to paediatric and adolescent HIV treatment, prevention, and service delivery. However, priority setting workshops focused on the needs of young people living with HIV are lacking in southern Africa. Here, we report the outcome of a priority setting workshop organised in Cape Town, South Africa with 19 young people living with HIV and their parents and caregivers. Workshops were facilitated by trained research and clinical staff, who provided a plain-language introduction to research questions for the attendees. During the day-long workshop, attendees developed a list of research questions concerning HIV-related physical health, mental health, and psychosocial support and later voted on the order of importance for the questions which they had collectively identified. Facilitators did not prompt any questions or amend the phrasing of questions generated by the attendees. A cure for HIV was highlighted as the most important research priority for young people living with HIV. Other priorities for young people included the effects of antiretroviral therapy on the body, the brain, and their social relationships, causes of emotional issues such as depression and mood swings, and potential interventions to reduce HIV-related stigma in schools through positive education for teachers and students. Research priorities for parents and caregivers included improving antiretroviral adherence through long-acting injections, mental health impacts of HIV status disclosure without consent, and improving support provided by local community clinics. The research questions identified through this workshop may be used by researchers to develop future studies which truly benefit young people living with HIV in South Africa and beyond.

## Introduction

The importance of taking lead from the community when designing, conducting, and disseminating biomedical research is increasingly being recognised [1]. Many institutions and funders now require that biomedical research should meaningfully involve people with relevant lived experience as early as possible, including when designing the study. This represents a paradigm shift away from *researcher-led research design*, where researchers identify questions and design studies with little to no input from people with lived experience, towards *community-led research design*, where community members take the lead on identifying questions they want to be answered and working with technical experts to develop research studies that address these priorities [2].

Priority setting workshops provide a space for community members to collectively identify these priorities and determine their order of importance, so that research may be best placed to truly benefit the community [3]. Large-scale priority setting workshops have previously been carried out to define priorities for HIV-related biomedical research for children and adolescents. These workshops, led by the World Health Organization (WHO) and the Collaborative Initiative for Paediatric HIV Education and Research (CIPHER) of the International AIDS Society (IAS), identified key research questions for HIV testing, treatment, and service delivery from respondents in over 60 countries [4, 5].

Although these large-scale prioritisation exercises offer value in identifying global trends in community priorities, understanding local community needs is equally important. Few published priority setting workshops have focused exclusively on people living with HIV in southern Africa–and none, to our knowledge, focusing on young people living with HIV–in part because these formal approaches to community engagement are relatively new in this region. Clarke and colleagues [6] recently reported on their priority setting exercise for older adults living with HIV in Tanzania, in which they noted that basic needs (income, nutrition, shelter) and removal of stigma were highly important to these individuals. The authors suggest that these priorities are aligned with the national context, including vulnerability to financial, food, and housing insecurity and increasing costs of healthcare visits. Therefore, priority setting workshops can offer crucial context for researchers who serve people living with HIV in resource-limited settings, but this context is currently missing as very few such workshops have been reported in southern Africa.

The Family Centre for Research with Ubuntu (FAMCRU) is a clinical research centre established in 2002 at Tygerberg Hospital in Cape Town, South Africa, which focuses on infectious disease research. We regularly recruit children and young people living with HIV for large-scale research studies and clinical trials, focused on HIV treatment and prevention, mental health issues, and psychosocial support for young people living with HIV and their caregivers. We also host monthly peer support workshops for young people who attend our clinic (regardless of HIV status) led by an experienced social worker (co-author TB). Here, we report on a priority setting workshop which we organised at FAMCRU, with the dual aim of enhancing trust and transparency with the community and taking lead from young people living with HIV on the research questions which we should pursue in the future.

## Methods

### Workshop attendees

We invited young people (aged 14 to 18 years old) living with perinatally-acquired HIV to this workshop, along with one parent or caregiver for each young person. Potential attendees were invited from amongst young people who lived in Cape Town and surrounding areas (primarily Khayelitsha) who had previously participated in research studies at FAMCRU. Attendees were invited to join the workshop regardless of their home language (English or isiXhosa). As this was a community engagement event and not a research study visit, we did not record any sociodemographic information for those who attended the workshop.

### Workshop structure

Young people living with HIV and their parents or caregivers attended separate, parallel workshops in different rooms so that all attendees could speak freely. The structure for both parallel workshops was harmonised. Both workshops were facilitated by research and clinical staff from FAMCRU, and there was at least one facilitator fluent in isiXhosa in both rooms. Instructions provided to workshop facilitators are available in **S1 File** for any researchers seeking to conduct similar workshops in future.

Workshops began with personal introductions and ground rules established by attendees and facilitators in each room. The facilitators provided a plain-language introduction to research questions, with some examples of questions which are currently being investigated at FAMCRU. The goal of the workshops was for attendees to identify research questions which matter to them around three themes: HIV-related *physical health*, *mental health*, and *psychosocial support*. Attendees in each room were split into three smaller discussion groups, in which they discussed each theme (in rotation) and developed research questions for it.

Following the small-group discussions, questions for each theme developed by all smaller groups were compiled. Attendees in each room then had an opportunity to revise or clarify the phrasing of any questions, after which all questions for each room were compiled into a ballot box. All attendees were then given a printed ballot paper and an equal number of stickers, which they could “spend” on questions according to the importance which they ascribed to each question, with a greater number of stickers assigned to a question indicating greater perceived importance. Votes for each question were then tallied from all attendees to develop a ranked list of research priorities. Throughout this process, questions developed by young people living with HIV and their parents or caregivers were kept separate.

Before adding questions to the ballot box, facilitators merged any questions from smaller groups which were identical or closely matched. Apart from this, the facilitators did not suggest any questions to attendees, nor did they amend or delete any questions identified by attendees.

### Ethical considerations

Community engagement exercises in the Global North are considered distinct from research. Participating in a research study, where a participant is told what to do by a researcher (passive participation) and is often exposed to novel or experimental procedures, is substantially different from participating in a community engagement event, where attendees (ideally) hold equal power to shape the direction of the event (active engagement) and risk of exposure to hazards is negligible. For these reasons, community engagement exercises in the Global North are often not subject to ethical review.

In southern Africa, where formal approaches to community engagement are newer, no standard guidance yet exists to inform ethical review for community engagement. Clarke et al. (2023) report, for instance, that their priority setting workshop underwent formal ethical review as a precautionary measure [6]. This lack of standard guidance may hinder good-faith efforts to create more opportunities for formal community engagement in the region, especially in comparison to the Global North. A summary of plans for our workshop were reviewed by leadership of the Stellenbosch University Health Research Ethics Committee (HREC), who confirmed that this event was distinct from research and thus did not require formal ethical review or informed consent from attendees.

We organised this workshop with several ethical considerations in mind. As we only invited young people living with HIV to the workshop, all potential attendees were informed that their participation in the workshop would indirectly disclose their HIV status to other attendees. To preserve attendees’ privacy, we did not request or record any personal identifiers or demographic information. No audio, photos, or video were recorded during the workshop. All attendees (young people and their parents or carers) were reimbursed for their time and their travel expenses to attend the workshop and lunch was provided.

## Results

The workshop was attended by 19 young people living with HIV, and their parents or caregivers. Each small discussion group thus had 6–7 attendees. Attendees were actively engaged throughout the day-long workshop and developed many potential research questions collectively. The full list of questions generated by young people and their parents and caregivers is available in **S2 File**. Note that we report questions generated by attendees verbatim, without editing any phrasing once these questions were approved by the attendees.

### Research priorities for young people living with HIV

The top five research priorities identified by young people living with HIV are shown in **Table 1**. These attendees collectively indicated that a cure for HIV remains the most important priority for young people living with HIV. Attendees also want to know effective ways of communicating one’s HIV status and whether an undetectable viral load allows them to engage in condom-less sex without the risk of HIV transmission to their partners. Possible links between antiretroviral therapy (ARVs) and depression were also identified. Finally, young people living with HIV in our workshop wanted to know whether their friends and family perceive them differently because of their HIV status (i.e. HIV-related stigma or discrimination).

**Table 1:** Top five research priorities identified by young people living with HIV.

Rank	Research Question
**1**	Can HIV be cured?
**2**	How can we communicate and disclose our HIV status to friends, family, and partners?
**3**	Is it safe to have unprotected sex when you are undetectable and your partner is HIV negative?
**4**	Do ARVs cause depression?
**5**	Do friends and family see us differently because of our HIV status?

ARV: antiretroviral therapy.

### Research priorities for parents and caregivers

Many parents were themselves living with HIV, so attendees in the parents and carers’ workshop also raised research questions that were relevant to their own experiences of living with HIV. The top five research priorities identified by parents and caregivers of young people living with HIV are shown in **Table 2**. These attendees want to know whether long-acting injectable ARVs can help improve their children’s adherence to ARV regimens. Attendees also expressed concerns about stigma at local community clinics, and wanted to know how these clinics can better support children living with HIV who have previously received their clinical care at specialist research clinics. Parents also identified gender-specific questions around their relationships with their children, including ways of empowering girls to make responsible sexual decisions as they approach young adulthood and improving communication between mothers and sons. Finally, parents and caregivers were interested in ways of encouraging family members to accept their children’s HIV status, “as much as [their child] has accepted this already.” (quote from anonymous workshop attendee)

**Table 2:** Top five research priorities identified by parents and caregivers of young people living with HIV.

Rank	Research Question
**1**	Can ARV injections help improve adherence?
**2**	How can local clinics better support children who are referred from research clinics?
**3**	How can we empower girls living with HIV to make responsible sexual decisions?
**4**	How can we improve relationships between mothers and sons?
**5**	How do we encourage family members to accept someone’s status?

ARV: antiretroviral therapy.

### Research priorities by theme

**Fig 1:**
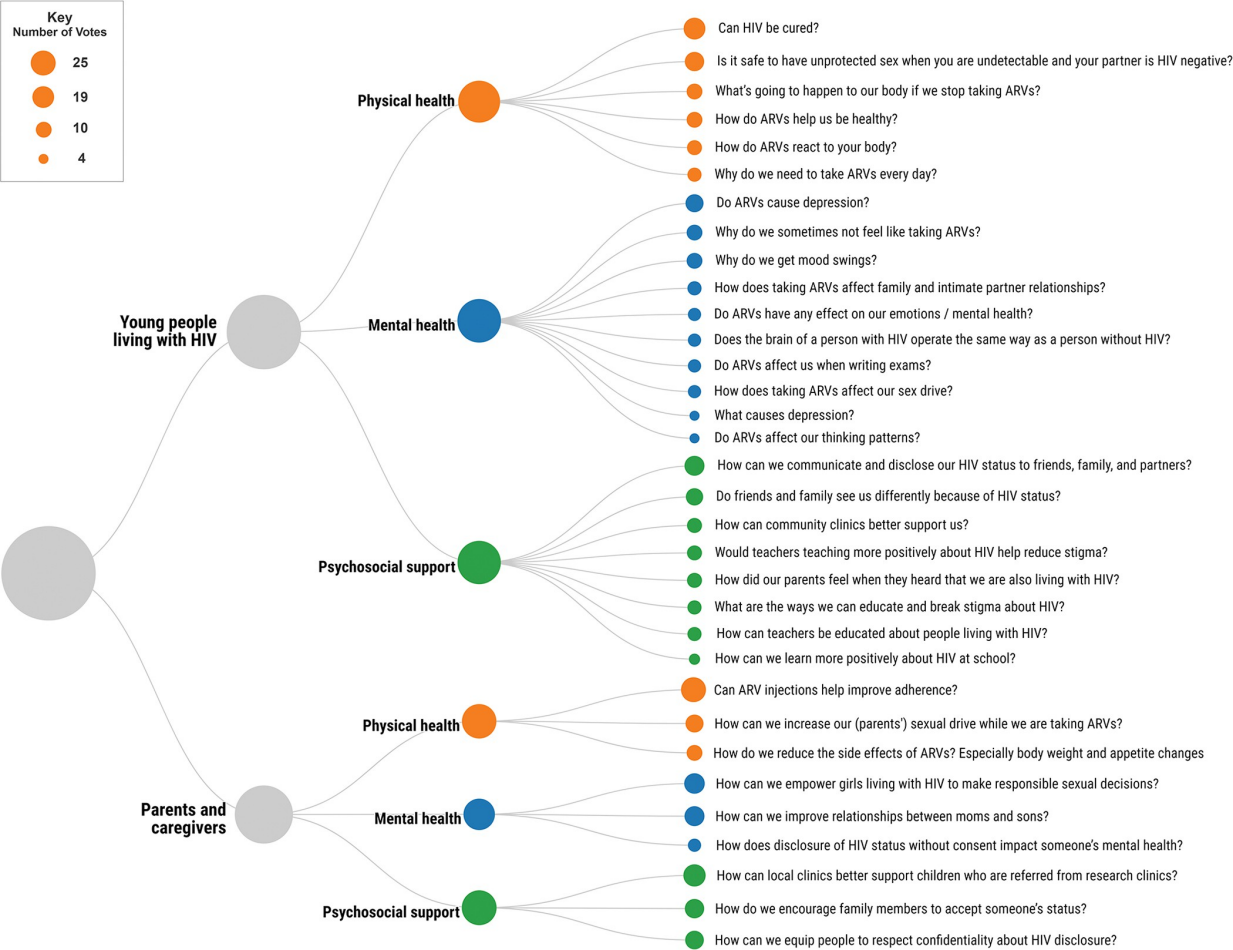
Top research priorities for young people living with HIV and their parents and caregivers for HIV-related physical health, mental health, and psychosocial support. The figure displays the top 5 ranked list of priorities for young people living with HIV, and the top 3 ranked list for their parents and caregivers; number of votes were tied for some questions identified by young people living with HIV. Size of each circle is proportional to the number of votes received for each question. Questions are displayed in descending order by number of votes within each category.

The top five research priorities identified by young people living with HIV, and top three research priorities identified by their parents and caregivers, ranked within each theme discussed in the workshop are shown in **Fig 1**. Parents and caregivers identified fewer questions (5–6 questions per theme) than young people living with HIV (10–12 questions per theme), so we highlight only the top three priorities for parents and caregivers in this figure, but the full list of questions identified by each group is available in **S2 File**.

For young people living with HIV, in addition to questions about a cure for HIV and risk of transmitting HIV when undetectable, the most important research priorities in HIV-related physical health revolved around ARVs. Attendees wanted to know how ARVs affect their bodies in positive or negative ways, the consequences of ARV interruption, and why ARVs must be taken daily. Potential consequences of taking ARVs were also highlighted in questions around mental health, such as why young people may not want to take ARVs and whether these medications affect their emotions, cognitive skills (e.g. exam-taking), relationships, or sex drive. Depression emerged as the top mental health research priority for young people living with HIV, with several highly-ranked questions revolving around the causes of depression, mood swings, and changes in emotional health. Notably, some (but not all) attendees had participated in depression-related research in 2022, so they may have been primed to thinking about depression as a primary concern. With regards to psychosocial support, young people living with HIV were most concerned with HIV status disclosure, perceived stigma or discrimination, and ways of positively educating both students and teachers at schools to reduce stigma.

For parents and caregivers of young people living with HIV, in addition to the potential benefits of long-acting injectable ARVs on treatment adherence, the top physical health-related priorities were potential side effects of ARVs, including changes in sex drive, body weight, and appetite (for their children as well as for themselves, in the case of parents who were also living with HIV). Parents and caregivers also prioritised research which may explain the mental health impacts of disclosure of one’s HIV status without one’s consent. In terms of psychosocial support, HIV status disclosure was highly ranked as a priority, with questions around encouraging others to accept someone’s status while also equipping them to treat such a disclosure with discretion and confidentiality.

## Discussion

We organised a priority setting workshop involving young people living with HIV, and their parents and caregivers, who reside in Cape Town, South Africa. Attendees at this workshop collectively identified a set of research priorities and voted to decide on their order of importance. Perhaps unsurprisingly, the research priority highlighted as the most important for young people in our workshop was a cure for HIV. These research priorities identified by attendees can inform future directions of research for biomedical scientists and clinicians serving young people with HIV. Many questions posed by young people and their carers may lead directly into future research, and indeed, several are already under investigation: for instance, reducing the side effects of ARVs on the body, health-related consequences of ARV interruptions, and possible differences in cognitive skills or brain function between people living with and without HIV. The research questions that emerged from our workshop, provided in **Tables 1** and **2 and Fig 1**, and **S2 File**, may be of broad interest to HIV researchers in South Africa and similar settings with high HIV prevalence.

Young people living with HIV and their parents and caregivers were particularly interested in how ARVs affect their health and their relationships. There were also overlapping interests in research on HIV status disclosure and stigma reduction between the two groups. Both groups also identified improved support from local community clinics for young people referred from research clinics as a priority. Given that the workshop attendees had previously received their HIV-related clinical care at FAMCRU, a specialist research clinic with staff who are trained to care appropriately for young people living with HIV, this research priority may indicate a gap in HIV-related sensitivity training in community clinics.

Notably, all young people who attended our workshop were living with perinatally-acquired HIV. While many of the questions identified by them could be relevant to other groups of young people living with HIV, those young people who acquire HIV later in life may have different priorities or may rank the importance of these priorities differently. Similarly, many parents of young people in our workshop were also living with HIV, whereas parents or caregivers of young people who acquire HIV later in life may not be living with HIV themselves and may propose different research priorities entirely. It will therefore be important to carry out similar workshops in future with other groups of young people living with HIV and their caregivers.

Notable differences between research priorities identified by young people and their carers included an emphasis amongst young people living with HIV on stigma reduction in school settings. These attendees spend a substantial proportion of their time in school, and many of their social relationships are also formed in this setting; thus, these young people highly prioritised research on positive education about HIV to help reduce stigma amongst both teachers and other students. Parents and caregivers were concerned about the mental health impacts of HIV status disclosure without consent on their children, as well as being interested in research on how they might emotionally prepare themselves for instances when their child’s status is disclosed without consent.

These priorities align with the local and national context, as young people living with HIV in South Africa continue to face substantial stigma and educational delays [7]. Discrimination due to HIV status is also a common experience amongst young people with HIV in South Africa [8]. For these reasons, young people living with HIV may emphasise the development of interventions to positively educate communities about HIV at school, whereas their parents and caregivers may prioritise research around HIV status disclosure.

The outcome of our priority setting workshop makes a compelling case for greater and more effective science communication by researchers serving young people living with HIV in South Africa. Certain research questions identified by young people in our workshop have already been investigated; in particular, there is robust evidence that people living with HIV who have an undetectable viral load cannot transmit the virus to their partner through condom-less sex [9]. Nevertheless, this was one of the top five research questions for young people in our workshop, which suggests that findings from emerging HIV-related biomedical research are not successfully reaching these young people (and, by extension, health educators). Thus, research findings must be shared by researchers and science communicators with these young people living with HIV more intentionally and effectively. Successful approaches may involve appropriate transcultural translation of dissemination materials and accessible social media formats which are most likely to reach young people living with HIV in resource-limited settings. Dissemination plans must meaningfully involve young people living with HIV in the respective local contexts to maximise the reach and impact of research findings.

Our priority setting workshop may offer a helpful model for other researchers serving people living with HIV in South Africa and beyond. Our event was well-attended and all attendees were actively engaged; this likely stemmed from existing positive relationships between the attendees and staff at FAMCRU who facilitated these workshops. All facilitators must be appropriately qualified to work with young people living with HIV and trained in the necessary facilitation skills. This is particularly important to avoid leading prompts and ensure that all questions are developed organically by the attendees. While we were successful in using stickers as votes which each attendee could “spend” to indicate their preferences, other researchers may try different ways of conducting voting to determine the order of importance for research questions. Activities within these workshops must be tailored to the language, literacy, and health literacy levels of all attendees. We found that inviting groups to designate one note-taker amongst themselves was a suitable way of ensuring that all groups recorded the questions they discussed, but that any attendees who were not comfortable with reading or writing were not excluded.

Finally, although similar priority setting workshops in southern Africa have undergone formal ethical review, we were successful in making a case to the relevant ethics review board that this activity did not require formal review. We encourage other researchers in southern Africa who wish to carry out similar priority setting workshops (while, of course, taking into consideration all notable ethical concerns for involving vulnerable individuals such as young people and those living with HIV) to use our work as an example when communicating with ethics review boards to determine eligibility for ethical review. This form of community engagement can be carried out ethically and responsibly without necessarily requiring formal ethical approval. By standardising this approach to the ethics surrounding community engagement, we may level the playing field for researchers in the Global South who are interested in community engagement practices but often have to complete additional administrative requirements compared to their colleagues in high-income countries.

We have shown here the feasibility of organising a small-scale priority setting workshop involving young people living with HIV and their parents and caregivers. Through this workshop, attendees developed a ranked list of research priorities which can inform future directions of inquiry for biomedical researchers serving young people living with HIV in South Africa and beyond. This workshop provides a model for meaningful engagement with people with relevant lived experience in a resource-limited setting, so that researchers take lead from the community–rather than assuming community needs–to inform future research design.

## Supporting information

S1 File Instructions provided to workshop facilitators. (DOCX)

S2 File Research priorities identified by young people living with HIV and their parents and caregivers. Number of votes received for each priority are provided next to each research question, along with a section rank (i.e. rank within the category in which the question falls) and an overall rank (i.e. rank across all three categories).
(XLSX)
