# Supplementary material for: Taking lead from the community: What do young people living with HIV want us to research?
Source: PLOS Glob Public Health. 2023 Dec 11;3(12):e0002605. doi: 10.1371/journal.pgph.0002605 (PMC10712858; doi:10.1371/journal.pgph.0002605)
Supplement: S1 File — (DOCX) [file pgph.0002605.s001.docx]

# Instructions to Workshop Facilitators

1. Personal introductions + ground rules (these apply to all attendees *and* facilitators)
2. People are welcome to step out to go to a quiet space if they need to at any point during the workshop
3. Words and stigma – what words we don’t want to be using – invite people to write words/phrases related to HIV we shouldn’t be using on a flip chart in the corner of the room; they can add to this throughout the day
4. Introduction to workshop
   1. What are research questions? – crucial to start with the same understanding
      1. questions that we can test in research, which may solve a problem faced by the community or help gather more information
   2. What kind of research do we currently do at FAMCRU? – give examples of *research questions* so people get a sense of what these typically look like
      1. Why are some people living with HIV more likely to get depression?
      2. Does HIV affect your thinking skills (cognition)?
      3. What effects does HIV have on your immune system?
   3. Goals of the workshop: **tell us what you think are important questions that we should be researching**
   4. Provide overview of structure and timing of workshop
5. Split into 3 groups, each to brainstorm questions on one theme (in rotation) – physical health, mental health, and psychosocial support
   1. Each group gets flip charts to write / take notes – one note-taker for each group nominated within the group
   2. One facilitator per small group – remind and encourage people to translate *needs* into *questions* – e.g. *I think we need more support from our school teachers* 🡺 *How can we increase support provided by school teachers for young people with HIV?*
   3. **Goal is for each small group to come up with at least 3 questions that are important to them for each theme**
   4. Questions shouldn’t be dismissed at this stage – even if one person is interested in something, it is worth noting – group priorities can emerge later
6. Return to full group to discuss any queries or concerns, wrap up discussions
7. Unstructured discussions for attendees to refine wording of questions or merge/unmerge questions
8. Facilitators create ballot paper for people to rank the questions in order of importance
9. 30 minutes for priority setting – everyone gets 10 stickers, and they can “spend” them on any of the priorities in any number/distribution they want to indicate what is important to them
10. Count up votes and rank research questions accordingly
11. Final queries, concerns, discussions
